# A quantitative model of water radiolysis and chemical production rates near radionuclide-containing solids

**DOI:** 10.1016/j.radphyschem.2015.06.011

**Published:** 2015-06-11

**Authors:** Mary E. Dzaugis, Arthur J. Spivack, Steven D'Hondt

**Affiliations:** Graduate School of Oceanography, University of Rhode Island, Narragansett Bay Campus, 215 South Ferry Road, Narragansett, RI 02882, USA

**Keywords:** Water radiolysis, Mathematical model, Dose rate, Hydrogen, Hydrogen peroxide, Spent nuclear fuel

## Abstract

We present a mathematical model that quantifies the rate of water radiolysis near radionuclide-containing solids. Our model incorporates the radioactivity of the solid along with the energies and attenuation properties for alpha (α), beta (β), and gamma (γ) radiation to calculate volume normalized dose rate profiles. In the model, these dose rate profiles are then used to calculate radiolytic hydrogen (H_2_) and hydrogen peroxide (H_2_O_2_) production rates as a function of distance from the solid–water interface. It expands on previous water radiolysis models by incorporating planar or cylindrical solid–water interfaces and by explicitly including γ radiation in dose rate calculations. To illustrate our model's utility, we quantify radiolytic H_2_ and H_2_O_2_ production rates surrounding spent nuclear fuel under different conditions (at 20 years and 1000 years of storage, as well as before and after barrier failure). These examples demonstrate the extent to which α, β and γ radiation contributes to total absorbed dose rate and radiolytic production rates. The different cases also illustrate how H_2_ and H_2_O_2_ yields depend on initial composition, shielding and age of the solid. In this way, the examples demonstrate the importance of including all three types of radiation in a general model of total radiolytic production rates.

## 1. Introduction

Water radiolysis is the dissociation of water molecules by ionizing radiation. Primary products of water radiolysis include several chemical species: e_aq_^−^, HO•, H•, HO_2_•, H_3_O^+^, OH^−^, H_2_O_2_ and H_2_ (Le Caër, 2011; Spinks and Woods, 1990). Given the potential reactivity of these primary products, water radiolysis is of interest in studies of many domains, including nuclear reactors (Burns et al., 2012), spent nuclear fuel (Jonsson et al., 2007), early Earth history (Draganić et al., 1991), and microbiology (Blair et al., 2007; Lin et al., 2005; Pedersen, 1996). In order to understand the importance of water radiolysis in these and other domains, accurate quantification of chemical production rates by radiolysis is vital. We present a new model to quantify the extent to which radiolysis occurs in water at phase boundaries where radioactive elements are present in a solid phase.

Multiple previous studies have examined product formation by water radiolysis at the interface between solids and water (e.g., Buck et al., 2012; Jonsson et al., 2007; Nielsen and Jonsson, 2006). In general, these studies have examined radiolysis associated with spent nuclear fuel. Experiments and kinetic modeling have focused on the impact of environmental parameters (such as pH, NaCl concentration and groundwater chemistry) on radiolytic production rates (Bruno et al., 2003; Ershov and Gordeev, 2008; Jonsson, 2012). Nielsen and Jonsson (2006) developed a geometric model that provides time extrapolations (100 y to 100 ky) of dose rates due to α and β radiation. Buck et al. (2012) used their model to examine how changing conditions, such as carbonate chemistry, brine concentration, and gas concentrations, affect the redox systems in water repositories.

Our study provides a general quantitative model for calculating radiolytic production rates as a function of distance from the solid–water interface. Our model differs from previous models in several respects. It differs from the models of Nielsen and Jonsson (2006) and Buck et al. (2012) by explicitly accounting for energy attenuation of α, β and γ radiation. It differs from the model of Nielsen and Jonsson (2006) by including γ radiation and by calculating the contribution from all radiation to the total absorbed dose. Our model also differs from previous models by explicitly considering both planar and cylindrical phase boundaries (previous models assumed planar boundaries) and by accounting for the extent of shielding material that surrounds a radioactive solid phase.

Here, we present the model and apply it to an example of spent nuclear fuel to highlight how the distributions of radiation-specific volume normalized dose rates and radiolytic production rates vary as a function of distance. In our example, we focus on radiation dose profiles and radiolytic H_2_O_2_ and H_2_ production profiles around spent fuel before and after barrier failure.

## 2. Methods

Each type of radiation, α, β and γ, has different radiolytic product yields. In addition, each follows a different attenuation law because α and β radiation behave as charged particles, while γ-rays have no charge or mass. Consequently, we use similar methods to calculate volume normalized dose rates for both α and β radiation, and a somewhat different method for γ radiation. (Throughout the remainder of this paper we refer to “volume normalized dose rate” simply as “dose rate”.) We first present our equations for calculating the dose rate of charged particles and then present the equations and additional geometric conditions needed to account for γ radiation. We then calculate radiolytic production rates based on the dose rate profiles.

### 2.1. Radiant flux and dose rate for α and β radiation

Nuclei emit α particles with specific kinetic energies, while β particles have a continuous spectrum of energies. Both α and β particles are emitted isotropically (Spinks and Woods, 1990). To assess the contribution of β particles, we take the average initial energy for each β decay as 1/3 of the maximum energy (L’Annunziata, 2007). Stopping distance (*R_stop_*) is the maximum distance traveled by charged particles; it is determined by initial energy and matrix composition (L’Annunziata, 2007). Since the distance traveled by charged particles in solids is relatively small, most solid–water interfaces can be assumed to be planar for α or β particles. For our study, if the stopping distance is less than the radius of curvature, we assume a planar boundary. If a radionuclide within the solid is at a greater depth from the surface than *R_stop_*, then the charged particle will not reach the solid–water interface. While 50% of the charged particles emitted by radio-nuclides located at the solid–water interface are directed into the water.

Determination of the total radiation energy reaching the water per area per time [the radiant flux density (*F*)] depends on

- power (*P*),
- irradiance (*I*), and
- attenuation (*a*).

Here, power (*P*) is the initial energy per unit time per solid angle associated with the radiation (kinetic energy for alpha and beta). It is determined by the decay energy and activity of each radionuclide. We calculate power for individual α and β particles by multiplying the radionuclide activity (*A*) for each radionuclide (*i*) by the initial radiation energy (*E*_0_) per decay (*j*) of the same radionuclide and dividing by 4*π* steradians (Eq. (1)),

(1) $$P(i,j)=\frac{A_{i}*E_{0(i,j)}}{4\pi }$$

**Fig. 1:**
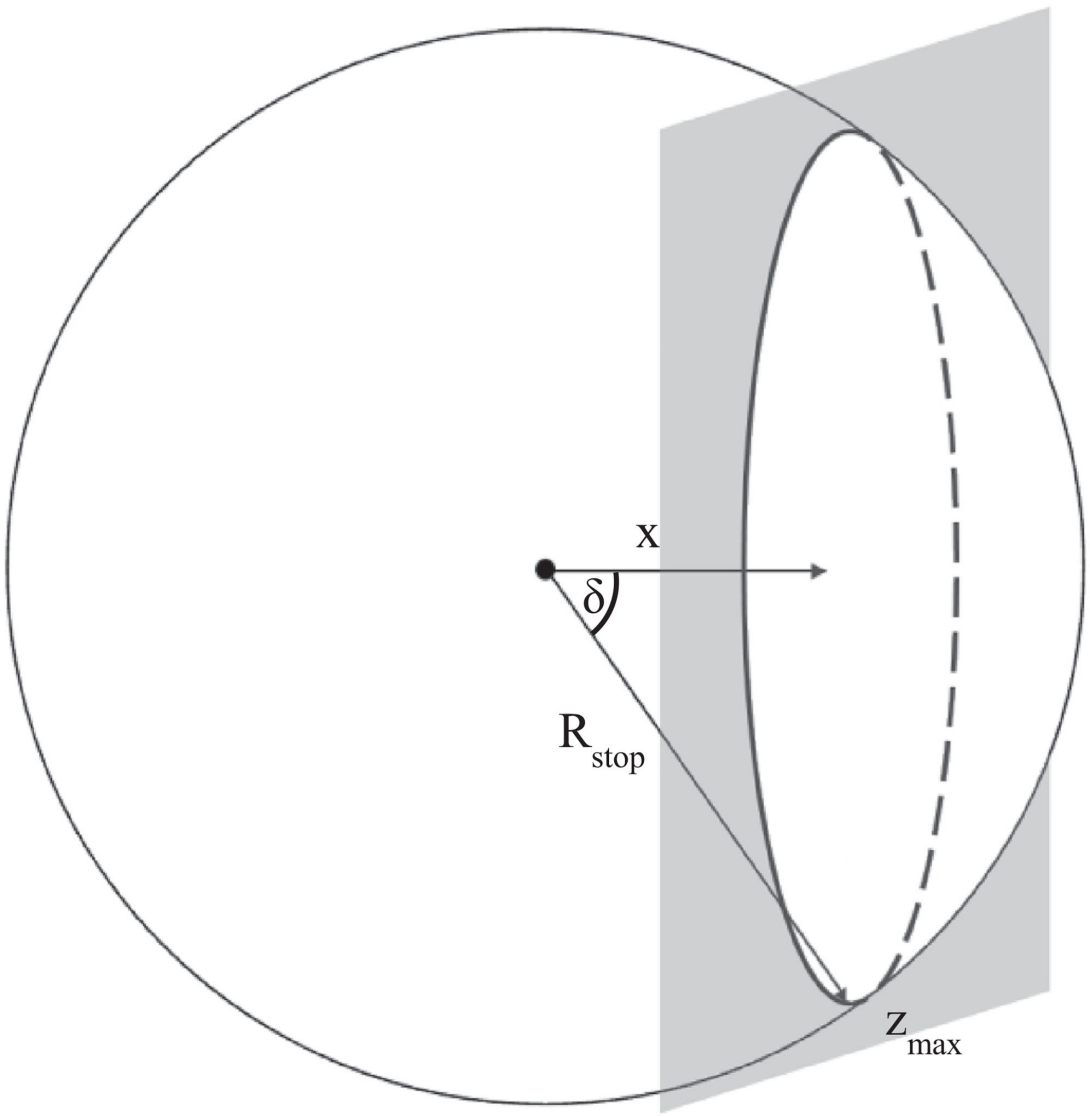
Schematic illustration for the α and β equations, depicting the path of isotropic radiation. The gray planar surface represents the solid–water interface (water to the right of the plane). *R_stop_* is the stopping distance of the traveling α or β particle, *x* is the distance from the interface where the radionuclide is located, and *δ* is the angle of incidence (the angle between particle's path and the normal to the planar interface).

The magnitude of *F* depends on the flux of radiant energy, *P*, per unit area [the irradiance, *I*]. How much radiation reaches the water depends on the particle's path to the solid–water interface. We assume the path is linear over the projected range along the initial travel direction. The irradiance (Eq. (2)) for each particle is determined by the angle of incidence (*δ*) and the inverse square of the distance traveled (*R*) (Fig. 1)

(2) $$I(i,j)=\frac{\text{cos }\delta }{{R_{\left(i,j\right)}}^{2}}$$

For both α and β particles, linear energy transfer (LET), energy attenuation per distance traveled, increases toward the end of the particle's path. We derive the energy remaining (*E_R_*) at distance R and the attenuation formula from Eq. (3), which is a simplification of an equation developed by Bethe and Ashkin (1953). Bethe's equation describes the relationship between energy and range of charged particles (Friedlander et al., 1964)

(3) $$\frac{\text{dE}}{\text{dR}}≅-\frac{d}{E}$$

The attenuation (*a*) is

(4) $$a(i,j)=\frac{E_{R}}{E_{0}}={\left(1-\frac{R}{R_{\text{stop}}}\right)}^{\frac{1}{b}}$$

where d and b are constants (Eqs. (3) and (4)). In the examples we present, the values for b and *R_stop_* are both determined using the experimental energy and range data from the ASTAR and ESTAR NIST databases (Berger et al., 2005) as well as the Range and Stopping Power application in Nucleonica (Nucleonica GmbH, 2014a). Although b has a value of 2 from Bethe's equation, experimentally its value varies. At lower initial energies, the dependence on energy is more closely proportional to *E*^3/4^, whereas at higher energies, it is better approximated by *E*^2^ (Alfassi and Peisach, 1991). To calculate b, we plot the energy versus projected range in log–log coordinates over the range of energies observed from the radioactive elements. The resulting slope is the value we use for b (Table A.2). We also determine *R_stop_* values using the energy-range data. The stopping distance depends on the initial energy of the particle and where in the path the charged particle crosses the solid–water interface. Multiple attenuation equations are used in the integration to account for the change of *b*-values in different materials (see Appendix for a more detailed discussion).

Combining equations for *P, I*, and *a*, and integrating throughout the radionuclide-containing solid, we calculate the total radiant flux density (*F_α,β_*). We sum over each radionuclide for all α and β decays with a unique *E*_0_ for the total radiant energy flux per radionuclide. We then sum the total radiant energy flux of all radionuclides to give the total radiant flux density,

(5) $$F_{\alpha ,\beta }=\sum_{i}\sum_{j}\int_{0}^{2\pi }\int_{0}^{R_{\text{stop}}}\int_{0}^{z_{\text{max}}}P(i,j)*I(i,j)*a(i,j)*z*\text{dz}*\text{dx}*d\theta$$

where *z_max_* depends on stopping distance and the distance of the radionuclide from the surface (see Eq. (A1.9) for expanded form). At any distance in the water from the surface, we calculate the absorbed dose rate (*D_α,β_*), based on *F* from Eq. (5). In our model, the absorbing volume is the water surrounding the solid. At any distance in the water, the average dose rate is equal to the radiant flux density divergence from solid–water interface divided by the distance in water.

### 2.2. Radiant flux and dose rate for gamma rays

We use similar methods to calculate the flux and dose rate for γ radiation. Gamma ray absorption, however, obeys an exponential law (the Beer–Lambert law) (Eq. (6)) characterized by an attenuation coefficient (*μ*).

(6) $$a_{\gamma }(i,j)=\frac{P_{R_{\gamma }}}{P_{0}}=e^{-\mu R_{\gamma }}$$

Attenuation coefficients are available for a wide range of elements and composite materials in the NIST X-ray Attenuation database (Hubbell and Seltzer, 2004). Different attenuation coefficients are used for each material through which γ-rays pass. In Appendix, we describe in detail how this is incorporated into the model. The ability of γ-rays to penetrate a specific matrix can also be described in terms of half-distances. The half-distance, *x*_½_ = 0.693/*μ*, is the thickness of material required to reduce the initial energy flux by one half (L’Annunziata, 2007). After 10 half-distances, slightly less than 0.1% of the initial radiation energy remains. To make sure that essentially all radiation is accounted for, we use 10 half-distances and operationally call this distance the maximum γ distance, *R_stop,γ_*.

Gamma rays have a smaller LET than charged particles. Consequently, their penetrating distance in a matrix is greater than α and β particles. The greater penetrating distance of γ radiation complicates modeling the radiolytic γ flux because possible curvature of the solid–water interface needs to be considered. Curved interfaces are often relevant for radiolysis studies. For example, spent nuclear fuel is typically a cylindrical pellet. For this study, we use cylindrical interface geometry when the radius of the cylinder is less than or equal to the maximum penetrating distance of the radiation. At these values, at least 20% of the radiation is not included in the radiant flux density calculations if a planar boundary rather than cylindrical is assumed.

**Fig. 2:**
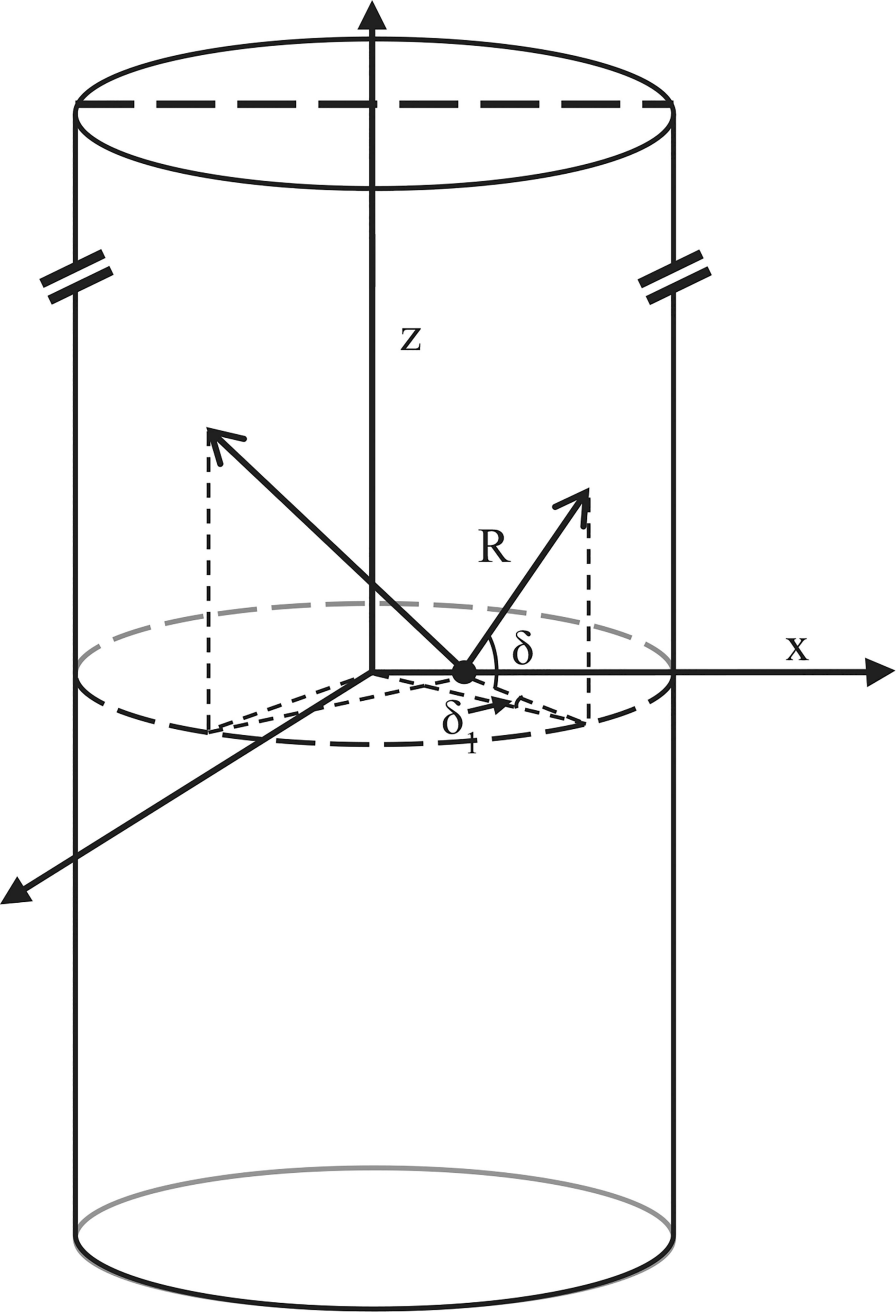
Representation of the possible paths of γ-rays emitted from a radionuclide (black dot) within a cylindrical solid. We assume the height, *z*, of the cylinder (typically a fuel rod) to be greater than the penetrating power of the γ radiation. *R* is the distance that the γ-ray travels in the solid. For the cylindrical boundary, the angle of incidence is a combination of *δ*_1_ and *δ*_2_.

Using a cylindrical solid–water boundary, the geometry changes the calculation of the γ-ray distance traveled, *R_γ_* (Fig. 2, Eq. (7)). We give the expansion of Eq. (7) in Appendix (Eq. (A2.4)).

(7) $$F_{\gamma }=\sum_{i}\sum_{j}\int_{0}^{2\pi }\int_{0}^{R_{{\text{stop}}_{\gamma }}}\int_{0}^{z_{{\text{max}}_{\gamma }}}P(i,j)*I(i,j)*a_{\gamma }(i,j)*z*\text{dz}*\text{dx}*d\theta$$

The equations for *P* and *I* are the same as those for α and β particles, however the equation for *a_γ_* is given by Eq. (6). Once *F_γ_* in known, we calculate the dose rate (*D_γ_*) due to γ radiation.

### 2.3. Radiolytic yields

The production rate of radiolytic products is the absorbed dose rate from each type of radiation multiplied by its respective *G*-value (the number of molecules created per 100 eV of energy) (Eq. (8))

(8) $$\text{Production rate}=(G_{\alpha }D_{\alpha }+G_{\beta }D_{\beta }+G_{\gamma }D_{\gamma })$$

*G*-values (*G_α,β,γ_*) depend on radiation type and differ for each radiolytic product. We list the *G*-values that we use for this study in Table 1.

### 2.4. Example

Since much radioactive waste is in the form of spent fuel assemblies, we use our model to calculate the radiolytic production distribution in water surrounding spent nuclear fuel. We chose this example because it illustrates the applicability of our method to calculate water radiolysis by γ radiation at a curved interface and the result has important implications for safe handling, disposal and storage of spent nuclear fuel. The dissolution rate of UO_2_ fuel is directly related to α, β and γ dose rates near the fuel surface. If the integrity of the barrier around a fuel pellet is breached and the barrier is infiltrated by water, radiation from the fuel will dissociate the water. Production of H_2_O_2_ can increase dissolution of UO_2_ in fuel pellets, while H_2_ suppresses dissolution by consuming H_2_O_2_ (Jonsson, 2012; Shoesmith, 2000, 2008).

For our example, we chose to use 20-year-old UO_2_ spent fuel with a 55 MWd/kgHM fuel burn-up. At this age, the fuel rod could still be stored in a spent-fuel pool, surrounded by water. The fuel rod contains stacked fuel pellets, which in our example are surrounded by Zircaloy cladding that creates a barrier for much of the radiation. We use nuclides that account for 99% of the radioactivity to calculate H_2_O_2_ and H_2_ production rates (Table A.1). WebKORIGEN was used to determine the activity of the radionuclides in the spent nuclear fuel (Nucleonica GmbH, 2014b). For this study, we assume the distribution of radionuclides within the pellet to be homogenous. In our example, we also assume the interface to be planar for α and β radiation because their maximum *R_stop_* in the fuel matrix (20 µm and 500 µm, respectively) is small compared to the curvature of the 1 cm diameter fuel rod. We test this assumption by comparing the area irradiated by α and β radiation using the curvature of the pellet to the area irradiated assuming a planar interface. We find that approximately 99% of the absorbed dose is accounted for when a planar surface is assumed.

We also assume constant *G*-values in our model and list the values we use in Table 1. For H_2_ yields, α radiation chemical yields have been shown to increase with the LET of the particle. However, for the energy range of α particles we are interested in this study (less than 5.8 MeV), LET appears to have a minimal effect on the *G*(H_2_) value. At high LET, *G*(H_2_) appears to plateau around 1.25 molecules/100 eV (Crumière et al., 2013). For H_2_O_2_ yields, Pastina and LaVerne (1999) also show that there is an increase in yield when LET increases. However, they show that an α particle with an LET more than 2 orders of magnitude greater than γ-rays has a H_2_O_2_ yield only 50% higher. The range of LET that we cover in our study, is much smaller than 2 orders of magnitude and therefore we assume constant *G*(H_2_O_2_) values. Pastina and LaVerne conclude that for heavy ions, the same H_2_O_2_ yields, within ±20%, can be used for a wide range of LET. We did not find any work specifically on the effect of β-particle LET, therefore we assume the H_2_ and H_2_O_2_ yields to be constant for *G_β_* as well.

We use our model to quantify the total dose rate and the radiolytic production of H_2_O_2_ and H_2_ as a function of distance from the solid–water boundary. It is important to note that these are production rates, not concentrations. We apply our model to the fuel rod before and after barrier failure, to compare α, β and γ distribution patterns about the fuel rod, and radiolytic production rates, under both conditions. The profiles also differentiate between the contributions of α, β and γ radiation to the dose and H_2_O_2_ and H_2_ production rates. Below, we describe the results of our model with a spent nuclear fuel example.

## 3. Results

**Fig. 3:**
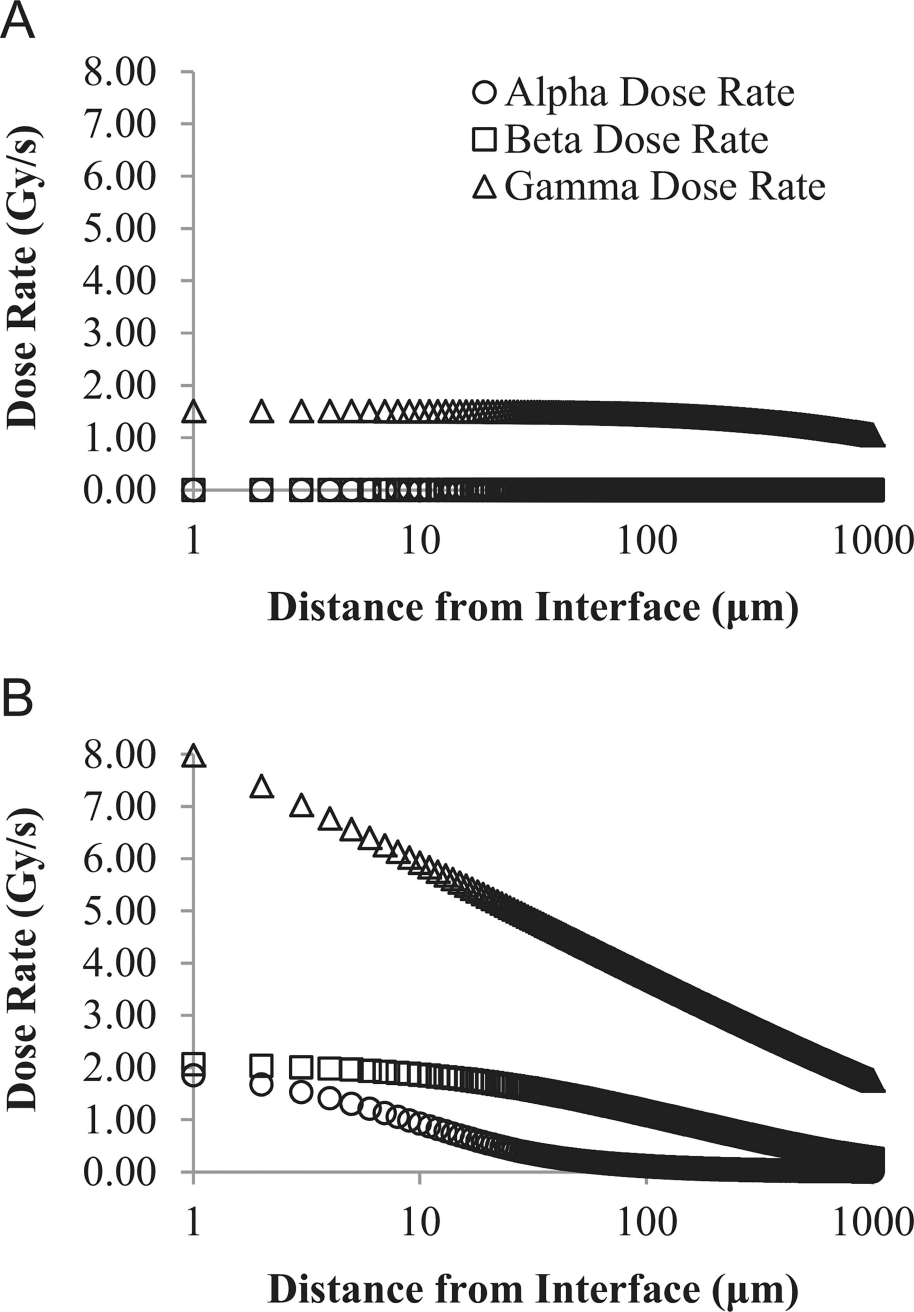
Calculated absorbed dose rate as a function of distance from the solid–water interface, with cladding intact (A) and after cladding failure (B) for 20-year-old fuel. Distance from pellet is plotted on a log scale. Dose rate from alpha and beta radiation in (A) are both 0 Gy/s.

We show the dose rate profiles from our 20-year-old spent nuclear fuel example in Fig. 3. This figure illustrates the absorbed dose rate for each type of radiation from 1 µm to 1000 µm from the fuel rod's surface. We show dose rate profiles for a fuel rod with intact cladding (Fig. 3A) and a fuel rod after cladding failure (Fig. 3B). The α and β dose rates are zero in Fig. 3A because the cladding stops all of the radiation from reaching the water (the cladding is thicker than the stopping distances for α and β radiation). However, γ radiation travels far enough to reach the water and *D_γ_* decreases away from the cladding–water interface. In Fig. 3B, α, β and γ radiation all are absorbed in the water, as there is no longer a barrier between the fuel surface and water. Dose rates decrease rapidly away from the surface interface. The dose rate due to γ radiation is approximately 5 times higher at the pellet–water interface then when the cladding is intact. For the 20-year-old fuel with barrier failure (Fig. 3B), *D_β_* is higher than the absorbed dose due to α particles. By 1000 µm, α and β particles only contribute about 17% to the average dose rate when barrier failure has occurred. Alpha particles emitted from the solid do not travel further than 50 µm away from the surface of the fuel, while γ radiation from the fuel pellet continues to be absorbed for tens of centimeters.

**Fig. 4:**
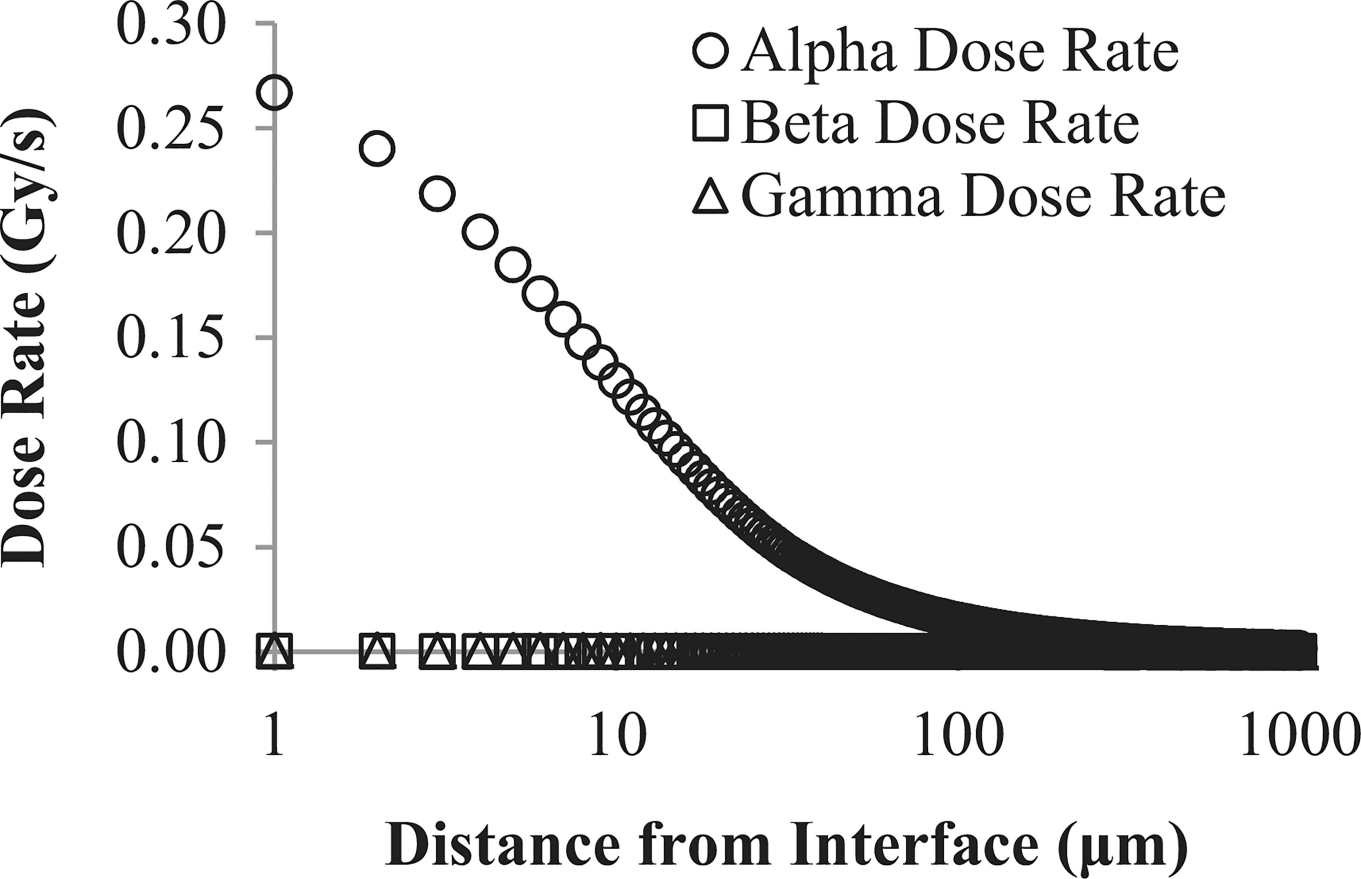
Dose rate profile for 1000-year-old fuel after cladding failure. Distance from interface is plotted on a log scale.

We also calculate the dose rate profile for 1000-year-old spent fuel with 55 MWd/kg burn-up after cladding failure (Fig. 4). Similar to Fig. 3, we show the dose rate profile for α, β, and γ radiation separately. At the surface of the 1000-year-old fuel, α-dose is responsible for over 99% of the total absorbed dose. The contribution to dose rate by β and γ radiation is non-zero, but very small. After 1000 µm, β-and γ-dose collectively account for 5% of the total dose of the entire volume. The γ-dose contribution is 4 times greater than β-dose at the fuel's surface. The total γ-dose over the whole 1000-µm interval is over an order of magnitude larger than the absorbed dose due to β radiation.

**Fig. 5:**
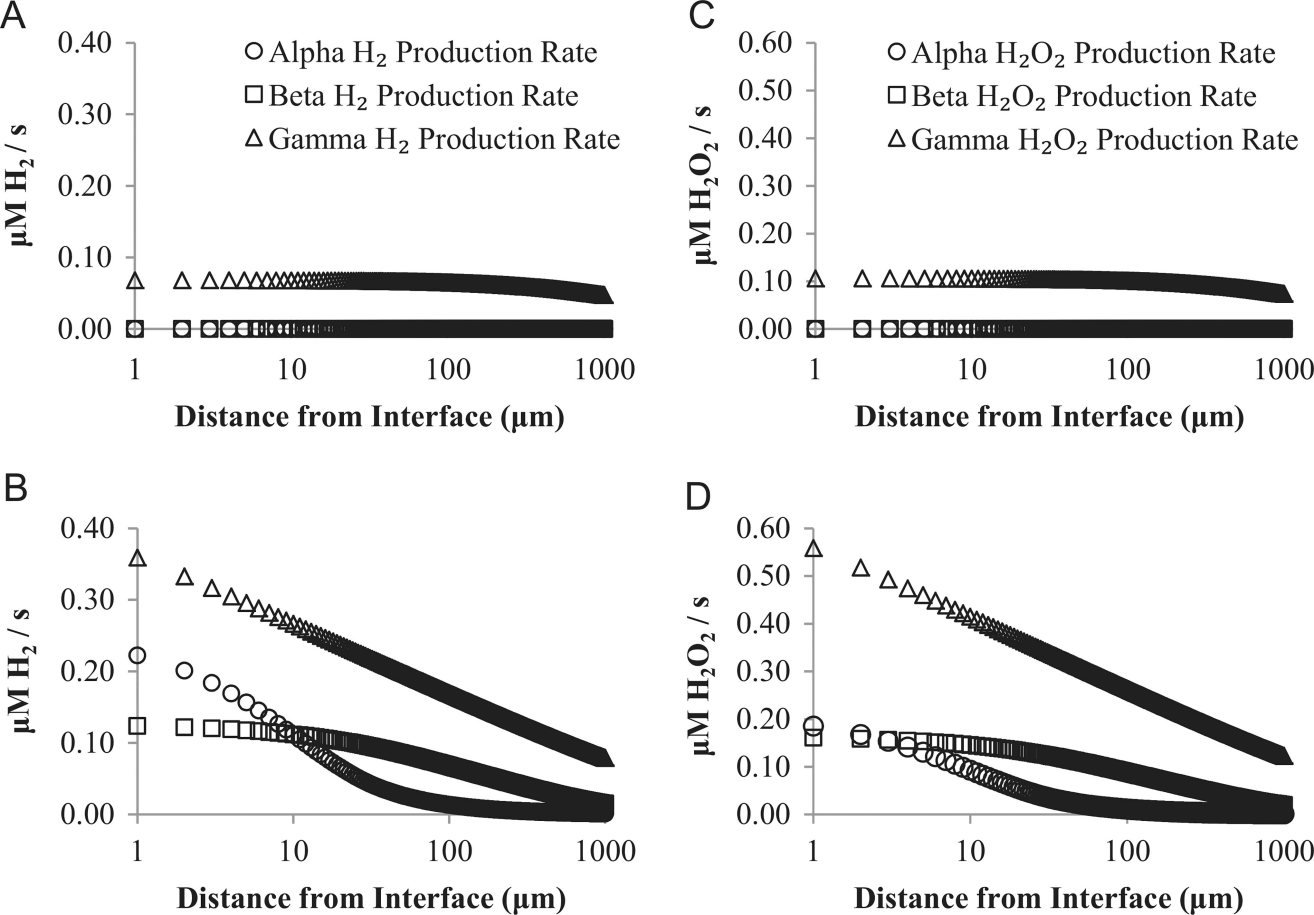
Radiolytic production rates for H_2_ before barrier failure (A) and after barrier failure (B). Production rates for H_2_O_2_ before barrier failure (C) and after barrier failure (D). All radiolytic production rates are for 20-year-old fuel. Distance from pellet is plotted on a log scale. Production rates from alpha and beta radiation in (A and C) are both 0 µM/s.

From the dose rates, we calculate radiolytic production rates (Fig. 5). Radiolytic production rates after barrier failure are almost an order of magnitude higher than before failure (Fig. 5). Total radiolytic H_2_ production rate is 10 times higher after barrier failure, while radiolytic H_2_O_2_ production increases 8.5 times. After barrier failure, α and γ radiation together contribute over 80% of radiolytic H_2_O_2_ and H_2_ production near the pellet–water interface. Alpha radiation contributes to water radiolysis only within 50 µm of the pellet. Farther from the interface, radiolytic production due to β and γ radiation dominates.

## 4. Discussion

Due to the high activity of young fuel assemblies and their close proximity to water in spent-fuel pools, application of our model to a 20-year-old fuel assembly nicely illustrates the model's capabilities. Typically, fuel assemblies are stored underwater in spent-fuel pools for up to 30 years to cool the fuel and provide shielding from radiation (IAEA, 1999). The radioactivity of spent fuel is highest during this time and α, β and γ radiation are emitted.

Hazards associated with spent fuel storage include release of radionuclides into the water or atmosphere and, in some cases, buildup of dangerous levels of H_2_ gas (Alvarez, 2011). The dissolution rate of the UO_2_ fuel matrix depends on the redox conditions at the fuel surface. Production of radiolytic oxidants (e.g., H_2_O_2_) and reductants (e.g., H_2_) directly influences UO_2_ dissolution (Burns et al., 2012). Previous models focused on the contribution of α and β radiation to radiolysis (i.e. Nielsen and Jonsson, 2006). However, in 20-year-old spent fuel, γ radiation significantly contributes to total dose rate (Fig. 3) and total radiolytic production rates (Fig. 5). Figs. 3 and 5 clearly show the importance of including γ radiation for this case. Gamma radiation in the fuel rod is predominantly produced by decay of ^137m^Ba. ^137m^Ba is a daughter of ^137^Cs, which has a 30-year half-life and the highest activity in 20-year-old fuel. Gamma rays from ^137m^Ba decay account for over 90% of the total gamma radiation emitted from spent fuel of this age. Although γ radiation has a lower *G*-value than α radiation (Table 1), the high activity of ^137m^Ba and long half-distances cause γ decay to be the principal cause of radiolytic H_2_ and H_2_O_2_ production in this example (Fig. 5).

We also use our model to examine radiolytic production rates while the Zircaloy cladding of the fuel assembly is intact (Fig. 5A,C), and after cladding failure (Fig. 5B,D). While the cladding is intact, only γ radiation can travel far enough to reach the water (Fig. 3A). However, if barrier failure occurs during storage in a spent-fuel pool, penetration of the water by α and β radiation increases total radiolytic production rates by almost an order of magnitude (Fig. 5) for both H_2_ and H_2_O_2_ production rates. This increase in radiolytic H_2_O_2_ production may increase the risk of UO_2_ dissolution.

These examples illustrate the importance of including γ radiation when quantifying radiolytic production rates, especially with young radioactive material. As radioactive material ages, γ radiation decreases and α-emitting radionuclides become relatively more important for radiolytic production. When spent fuel is older, α radiation dominates radiolytic production near the solid–water interface (Fig. 4). Since α-dose is the largest contributor to the total dose, our model produces a similar dose rate, for the 1000-year-old example within the range of α particles, to those calculated by Nielsen and Jonsson (2006). Although the contribution of β and γ radiation to dose rate is relatively small, γ radiation accounts for a larger percent of the total dose rate than β radiation. In short, the importance of γ radiation for radiolytic production rates relative to α and β radiation depends on spent-fuel age. In all cases, its inclusion provides a more complete and accurate understanding of the distribution of radiolytic products.

As stated in our Methods, for this study, we assumed homogenous distribution of radionuclides throughout the solid. This is an ideal case. For example, Burns et al. (2012) showed enrichment in Pu on the rim of fuel pellets. Our model can be adapted to include different zones of activity within the solid, which will produce different radiolytic production profiles.

## 5. Conclusion

We present a general model for quantifying water radiolysis by α, β and γ radiation near solid-water interfaces. Our model includes explicit consideration of the radiation's energy attenuation. It can be applied to different radionuclide containing materials, such as solid radioactive waste as well as naturally occurring rocks. By incorporating the activity, irradiance, and attenuation of radiation, our model separately calculates radiolysis due to α, β and γ radiation as a function of distance from the solid surface. As an example, we calculate total dose rates and radiolytic production rates for spent fuel to illustrate the importance of including the contribution from all three types of radiation in a general model of water radiolysis. While α radiation dominates radiolysis near the surface of old (1000-year-old) spent fuel with breached cladding, β and γ radiation contributes greatly to chemical radiolytic production from young (20-year-old) spent fuel with breached cladding. In the young fuel, γ radiation dominates radiolytic chemical production adjacent to spent fuel with intact cladding.

## Figures and Tables

**Table 1:** *G*-values (µmol/J) used in model calculation.

	H_2_	H_2_O_2_
*G_α_*	0.12^a^	0.10^a^
*G_β_*	0.06^b^	0.078^c^
*G_γ_*	0.045^a^	0.07^a^

a Pastina and LaVerne (2001).

b Kohan et al. (2013).

c Mustaree et al. (2014).

## Appendix

### Derivation of model equations

Definitions of variables used in radiant flux density calculation:

*D*=Dose rate (Gy s^−1^)
*F_α,β_*=radiant flux density (J cm^−1^ s^−1^)
*P*=power, energy released per time per solid angle (MeV s^−1^ sr^−1^)
*A*=activity (Bq g^−1^)
*E*_0_=initial radiation energy (MeV decay^−1^)
*I*=irradiance, power (*P*) per unit area (µm^−2^)
*δ*= angle of incidence on the irradiated surface (rad)
*R*= distance to the boundary, assumed linear over the projected range along the initial direction of the emitted particle (µm)
*R_γ_*= distance to the cylindrical boundary for γ-rays (µm)
*R_stop_*=stopping distance of a charged particle, maximum travel distance determined by the initial energy of the charged particle (µm)
*R*_0_=diameter of a cylindrical solid (µm)
*μ*=attenuation coefficient for gamma radiation (µm^−1^)
*x*=distance from the surface to a radionuclide of interest within the solid (µm)
*z*=distance irradiated along planar or cylindrical surface, where *z*=0 is perpendicular to the radionuclide source and *z_max_*=*R_stop_* (µm)
*w*=distance in water the from solid–water interface (µm)

#### A1 Radiant flux for α and β particles

##### A1.1 Equations used in F_α,β_ calculation (as a function of x and z)

- Power
  (A1.1) $$P(i,j)=\frac{A_{i}*E_{0(i,j)}}{4\pi }$$
  The initial radiation energy (kinetic energy for α and β and electromagnetic for γ radiation), *E*_0_, includes the parent–daughter nuclide branching fraction and the α and β branching intensity. The branching intensity values are based on the frequency that particular *E*_0_ values occur per 100 parent decays. We calculate *E*_0_ with values from the Nuclide Datasheets in Nucleonica (Nucleonica GmbH, 2014c). The isotopes used for the example calculations are listed in Table A.1. These isotopes account for 99% of the radioactivity within the pellets. The activity data is from WebKORIGEN (Nucleonica GmbH, 2014b).
- Irradiance (Eq. (A1.2)) determines how *P* changes as a function of the angle of incidence and the distance radiation has traveled.
  (A1.2) $$I=\frac{\text{cos }\delta }{R^{2}}$$
  Substituting $\text{cos }\delta =\frac{x}{R}$ and $R=\sqrt{{\left(x+w\right)}^{2}+z^{2}}$ gives irradiance at a specific distance from the interface, *w*, in terms of *x* and *z* (Eq. (A1.3)):
  (A1.3) $$I=\frac{x}{{\left({\left(x+w\right)}^{2}+z^{2}\right)}^{3/2}}$$
  **Table A.1:** List of isotopes.
  20-year-old pellets	1000-year-old pellets
  ^137^Cs	^241^Am
  ^137^mBa	^240^Pu
  ^241^Pu	^239^Pu
  ^90^Y	^235^mU
  ^90^Sr	^243^Am
  ^238^Pu	^239^Np
  ^85^Kr	^99^Tc
  ^244^Cm	
  ^241^Am	
  ^154^Eu	
  As a test case, we calculate the flux, *F*, for a non-attenuated radioactive source at any depth, *x*. The total power flux through an infinite planar boundary is described by:
  $$F=\int_{0}^{2\pi }\int_{0}^{\infty }P*I*z*\text{dz}*d\theta =\frac{P}{2}$$
  Half of the power reaches the solid–water boundary regardless of the location of the radioactive source. This is what we expect with no attenuation of the particles. However α and β particles lose energy as they travel, which reduces the total flux.
- Attenuation

Theoretically, energy loss is inversely proportional to the particle's energy (Bethe and Ashkin, 1953). We base derivation of the attenuation equation (Eq. (A1.5)) on a simplification of Bethe's formula, which describes the energy loss of a particle traveling through matter,

(A1.4) $$\frac{\text{dE}}{\text{dr}}≅-\frac{d}{E}$$

where d is constant for a constant travel matrix. However, experimentally this value depends on *E*_0_ of the charged particle. For the derivation of the attenuation equation, we integrate *E*^*b*−1^ to assign more correct values to the attenuation equation by using *E*_0_ and range relationships to determine a value for b. We calculate the fraction of initial energy remaining at distance *R* (*E_R_*) by integrating Eq. (A1.4).

$$\int_{0}^{R}\text{dr}=\int_{E_{0}}^{E_{R}}\frac{-E^{b-1}}{d}\text{dER}=\frac{{E_{0}}^{b}}{d*b}-\frac{{E_{R}}^{b}}{d*b}R_{\text{stop}}=\frac{{E_{0}}^{b}}{d*b}$$

Therefore, the fraction of the total stopping distance a particle travels at some distance *R* is

$$\frac{R}{R_{\text{stop}}}=1-\frac{E_{R}^{b}}{E_{0}^{b}}$$

The attenuation, or fraction of initial energy that reaches some distance *w* into the water, is described by Eq. (A1.5) or in terms of *x* and *z* as Eq. (A1.6),

(A1.5) $$a=\frac{E_{R}}{E_{0}}={\left(1-\frac{R}{R_{\text{stop}}}\right)}^{\frac{1}{b}}$$

Substituting $R=\sqrt{{\left(x+w\right)}^{2}+z^{2}}$,

(A1.6) $$a={\left(1-\frac{\sqrt{{\left(x+w\right)}^{2}+z^{2}}}{R_{\text{stop}}}\right)}^{\frac{1}{b}}$$

The attenuation equation (Eq. (A1.6)) depends on the particle energy and material. Using the energy-range data, we determine b and *R_stop_* by fitting an equation to the data over the energy ranges emitted by the radioisotopes. Table A.2 summarizes the values we use in this study. We calculate the particle's travel range (µm) for the energy ranges indicated in Table A.2 with the formula $R=h*E_{0}^{b}$. The values of b used in this study are comparable to those used in other studies. For example, the travel range we calculate for the α particles emitted in our UO_2_ examples are within 7% of those calculated using the equation presented in Nielsen and Jonsson (2006).

**Table A.2:** Energy-range equations and *b*-values used in calculations.

Radiation type	Material	Energy range (MeV)	*h*	b- Value
Alpha	UO_2_^a^	3.00–9.00	1.275	1.39
	Water^b^	2.00–9.00	3.627	1.47
		< 2.00	5.490	0.81
Beta	UO_2_^b^	1.00E-02–4.50E-01	1.00E03	1.58
		4.50E-01–4.50E00	6.63E02	1.07
	Water^b^	1.00E-02–4.50E-01	6.69E03	1.70
		4.50E-01–4.50E00	4.19E03	1.17

a Data from Nucleonica Range and Stopping Power application using a user defined compound of UO_2_ (Nucleonica GmbH, 2014a).

b Data from NIST ASTAR and ESTAR databases (Berger et al., 2005).

*R_stop_* is calculated by determining the distance radiation travels through the pellet matrix (or cladding) before reaching the water. If *R_stop_* is greater than the distance to the interface then the energy remaining, *E_R_*, is calculated (Eq. (A1.5)) and used as the initial energy for calculating how far the particle will penetrate the water. We then use this distance to calculate the new attenuation equation of the particle through water. Two different attenuation equations are included in the final integration, in our case one for the pellet and one for the water.

##### A1.2 F_α,β_ calculation

To calculate the total radiant flux density that reaches the solid–water boundary, we integrate the equations presented above over the depth of the radionuclide-containing solid, *x_max_*, and over the irradiated area of the planar boundary (Eq. (A1.7)). We can calculate the flux for any distance from the solid–water interface.

(A1.7) $$F_{\alpha ,\beta }=\int_{0}^{\theta_{\text{max}}}\int_{0}^{x_{\text{max}}}\int_{0}^{z_{\text{max}}}P(i,j)*I(i,j)*a(i,j)*z*\text{dz}*\text{dx}*d\theta$$

where *z_max_* depends on stopping distance of the particle through water, *R_water_*, the depth of the radionuclide, and the distance in water from the solid–water interface (*w*) (Eq. (A1.8)). *x_max_* is the depth at which only the most energetic α or β particle reaches the solid–water boundary (the greatest depth within the solid where particles can reach the water) and *θ* varies from 0 to 2*π*. We calculate the total radiant flux density by integrating and summing over all α and β particles and radionuclides (Eq. (A1.9)).

(A1.8) $$x_{\text{max}}=\sqrt{R_{\text{water}}^{2}-{\left(x+w\right)}^{2}}$$

(A1.9) $$F_{\alpha ,\beta }=\sum_{i}\sum_{j}\int_{0}^{2\pi }\int_{0}^{x_{\text{max}}}\int_{0}^{\sqrt{R_{\text{water}}2-{\left(x+w\right)}^{2}}}P(i,j)*I(i,j)*a(i,j)*z*\text{dz}*\text{dx}*d\theta$$

#### A2. Radiant flux density for gamma radiation

Due to the greater penetrating power of gamma rays, the cylindrical shape of the fuel rod needs to be considered. While this does not affect the overall structure of the flux equation, it does change the expression for *R_γ_* in terms of *x* and *z*. In the following equations, *θ* is the angle of rotation about the *z*-axis.

##### A2.1 Equations used in F_γ_ calculation (in terms of x, θ and z)

- Power, same calculation used for α and β particles (Eq. (A1.1))
- Irradiance for γ-rays and cylindrical pellet (Eq. (A2.2), Fig. 2 for illustration of angles *δ*_1_ and *δ*_2_)
  (A2.1) $$I_{\gamma }=\frac{\text{cos }\delta_{1}*\text{ cos }\delta_{2}}{R_{\gamma }^{2}}$$
  where $\delta_{1}={\text{tan}}^{-1}(\frac{R_{0}*\text{ sin }\theta }{R_{0}*\text{ cos }\theta -x})-\theta$ for $0\leq \theta \leq {\text{cos}}^{-1}(\frac{x}{R_{0}}),\delta_{1}=\pi -{\text{tan}}^{-1}(\frac{R_{0}*\text{ sin }\theta }{R_{0}*\text{ cos }\theta -x})-\theta$ for ${\text{cos}}^{-1}(\frac{x}{R_{0}})\leq \theta \leq \pi ,\text{cos }\delta_{2}=\frac{r\prime }{R_{\gamma }}$,
  $$r\prime =\sqrt{{\left(R_{0}+w\right)}^{2}-2(R_{0}+w)x\text{ cos }\theta +x^{2}}$$
  and $R_{\gamma }=\sqrt{r^{\prime 2}+z^{2}}$,
  (A2.2) $$I_{\gamma }=\frac{\text{cos }\delta_{1}*r\prime }{R_{\gamma }^{3/2}}$$
- Attenuation of γ-rays (Eq. (A2.3))
  (A2.3) $$a_{\gamma }=\frac{P_{R}}{P_{0}}=e^{-\mu R_{\gamma }}$$

We use specific *μ* values to calculate the attenuation through the solid and water, to get the total attenuation of the γ-rays at some distance, *w*. The attenuation coefficients are from the X-ray attenuation database (Hubbell and Seltzer, 2004). To account for different materials γ-rays encounter, each material has its own attenuation equation. For example, if a γ-ray was to pass through the pellet and then water, the total attenuation equation would be *a_γ_* = *e*^−*μ_pellet_R_pellet_*^ **e*^−*μ_water_R_water_*^. Where *μ_pellet_* and *μ_water_* are the attenuation coefficients for γ radiation in the pellet and water, respectively and *R_pellet_* and *R_water_* is the distance the γ-ray traveled through the pellet and water, respectively. These distances are calculated in a similar way to the *R_γ_* however the distance is divided into the pellet and water components.

##### A2.2 F_γ_ calculation

*F_γ_* calculation is very similar to charged particles. However, we divide the integral into two parts to account for the geometric effects on *δ*_1_ and *δ*_2_.

(A2.4) $$F_{\gamma }=\sum_{i}\underset{j}{\sum 2}\int_{0}^{z_{\text{max}}}\int_{0}^{x_{\text{max}}}\int_{0}^{{\text{cos}}^{-1}(\frac{x}{R_{0}})}P(i,j)*I_{\gamma }(i,j)*a_{\gamma }(i,j)*z\;*d\theta *\text{dx}*\text{dz}\;+2\int_{0}^{z_{\text{max}}}\int_{0}^{x_{\text{max}}}\int_{{\text{cos}}^{-1}(\frac{x}{R_{0}})}^{\pi }P(i,j)*I_{\gamma }(i,j)*a_{\gamma }(i,j)*z*d\theta \;*\text{dx}*\text{dz}$$

where *x_max_* is equal to radius of the cylinder, *R*_0_, and *z_max_* is equal to 10 times the half-distance of the γ-ray.

#### A3. Volume normalized dose rate calculation

The volume normalized dose rate calculation is described by Eq. (A3.1). The dose rate is calculated per volume of water surrounding the pellet, as thick as the *w* of interest. *F_α,β,γ_* is in units of J µm^−1^ s^−1^. By multiplying by the density of water, *ρ_water_*, the dose rate can be converted into Gy s^−1^

(A3.1) $$D_{\alpha ,\beta ,\gamma }=\frac{F_{w}-F_{\text{interface}}}{w}*\rho_{\text{water}}$$
